# A rapid screening classifier for diagnosing COVID-19

**DOI:** 10.7150/ijbs.53982

**Published:** 2021-01-09

**Authors:** Yang Xia, Weixiang Chen, Hongyi Ren, Jianping Zhao, Lihua Wang, Rui Jin, Jiesen Zhou, Qiyuan Wang, Fugui Yan, Bin Zhang, Jian Lou, Shaobin Wang, Xiaomeng Li, Jie Zhou, Liming Xia, Cheng Jin, Jianjiang Feng, Wen Li, Huahao Shen

**Affiliations:** 1 Key Laboratory of Respiratory Disease of Zhejiang Province, Department of Respiratory and Critical Care Medicine, Second Affiliated Hospital of Zhejiang University School of Medicine, Hangzhou, Zhejiang, China.; 2 Department of Automation, Tsinghua University, Beijing, China.; 3 Radiation Oncology, Stanford University School of Medicine, Stanford, CA, USA.; 4 Department of Respiratory Disease, Tongji Medical College, Huazhong University of Science and Technology, Wuhan, China.; 5 Department of Radiology, Second Affiliated Hospital of Zhejiang University School of Medicine, Hangzhou, Zhejiang, China.; 6 Department of Radiology, Tongji Medical College, Huazhong University of Science and Technology, Wuhan, China.

**Keywords:** COVID-19, chest X-ray, clinical feature, deep learning

## Abstract

**Rationale:** Coronavirus disease 2019 (COVID-19) has caused a global pandemic. A classifier combining chest X-ray (CXR) with clinical features may serve as a rapid screening approach.

**Methods:** The study included 512 patients with COVID-19 and 106 with influenza A/B pneumonia. A deep neural network (DNN) was applied, and deep features derived from CXR and clinical findings formed fused features for diagnosis prediction.

**Results:** The clinical features of COVID-19 and influenza showed different patterns. Patients with COVID-19 experienced less fever, more diarrhea, and more salient hypercoagulability. Classifiers constructed using the clinical features or CXR had an area under the receiver operating curve (AUC) of 0.909 and 0.919, respectively. The diagnostic efficacy of the classifier combining the clinical features and CXR was dramatically improved and the AUC was 0.952 with 91.5% sensitivity and 81.2% specificity. Moreover, combined classifier was functional in both severe and non-serve COVID-19, with an AUC of 0.971 with 96.9% sensitivity in non-severe cases, which was on par with the computed tomography (CT)-based classifier, but had relatively inferior efficacy in severe cases compared to CT. In extension, we performed a reader study involving three experienced pulmonary physicians, artificial intelligence (AI) system demonstrated superiority in turn-around time and diagnostic accuracy compared with experienced pulmonary physicians.

**Conclusions:** The classifier constructed using clinical and CXR features is efficient, economical, and radiation safe for distinguishing COVID-19 from influenza A/B pneumonia, serving as an ideal rapid screening tool during the COVID-19 pandemic.

## Introduction

Coronavirus disease 2019 (COVID-19) caused by severe acute respiratory syndrome coronavirus 2 (SARS Cov-2) first reported in December 2019 and has caused a global pandemic. Up to now, more than 61 million infected patients were reported globally, with 1.4 million deaths.

The diagnosis of COVID-19 is challenging in many countries due to its nonspecific symptoms and variable incubation period. Clinically, patients with COVID-19 can have presentations ranging from asymptomatic to severely ill. The most common symptoms are fever, fatigue, dry cough, myalgia, and dyspnea 1-5, while less common symptoms include diarrhea, hemoptysis, and headaches 3-5. These clinical manifestations are to some degree identical to those of other known pneumonias, especially influenza.

It is important to devise a rapid, economical, accurate approach to identify and diagnose the suspected population with COVID-19. The reference standard procedure for confirming the diagnosis is reverse transcriptase-polymerase chain reaction (RT-PCR) 5. However, debates remained due to varied sensitivity of RT-PCR 3-6.

Recent studies have suggested that chest computed tomography (CT) can be used as a screening and diagnostic tool in epidemic areas with the sensitivity up to 97% 5-7. However, several factors are concerns, delayed use because it is a limited medical resource during the COVID-19 pandemic, financial burden, the labor involved, difficulty disinfecting the device 5.

Deep learning has been widely used in medical image analysis, and the application of artificial intelligence (AI) to the diagnosis of COVID-19 has been proposed 8. Trained with much labeled data, several deep learning systems were shown to be more accurate than human radiologists at identifying COVID-19 9, 10. Most of these findings were based on CT information, and the performances were consistently satisfactory 11-16. Given its restricted availability, higher radiation exposure, and complicated disinfection procedures, alternative approaches to chest CT are needed and chest x-ray (CXR) diagnosis in COVID-19 should be examined. Compare to chest CT, CXR is a simple procedure with lower cost and radiation exposure. Several studies proposed that CXR imaging can be used for the early diagnosis of COVID-19 17, 18, but the reported accuracy was inferior to that of chest CT 19, 20. Therefore, the current study used deep learning methods to construct a classifier combining clinical features with CXR information as a simple, efficient, economical, and accurate approach to differentiate COVID-19 from influenza A/B.

## Methods

### Patients and Materials

**Figure 1:**
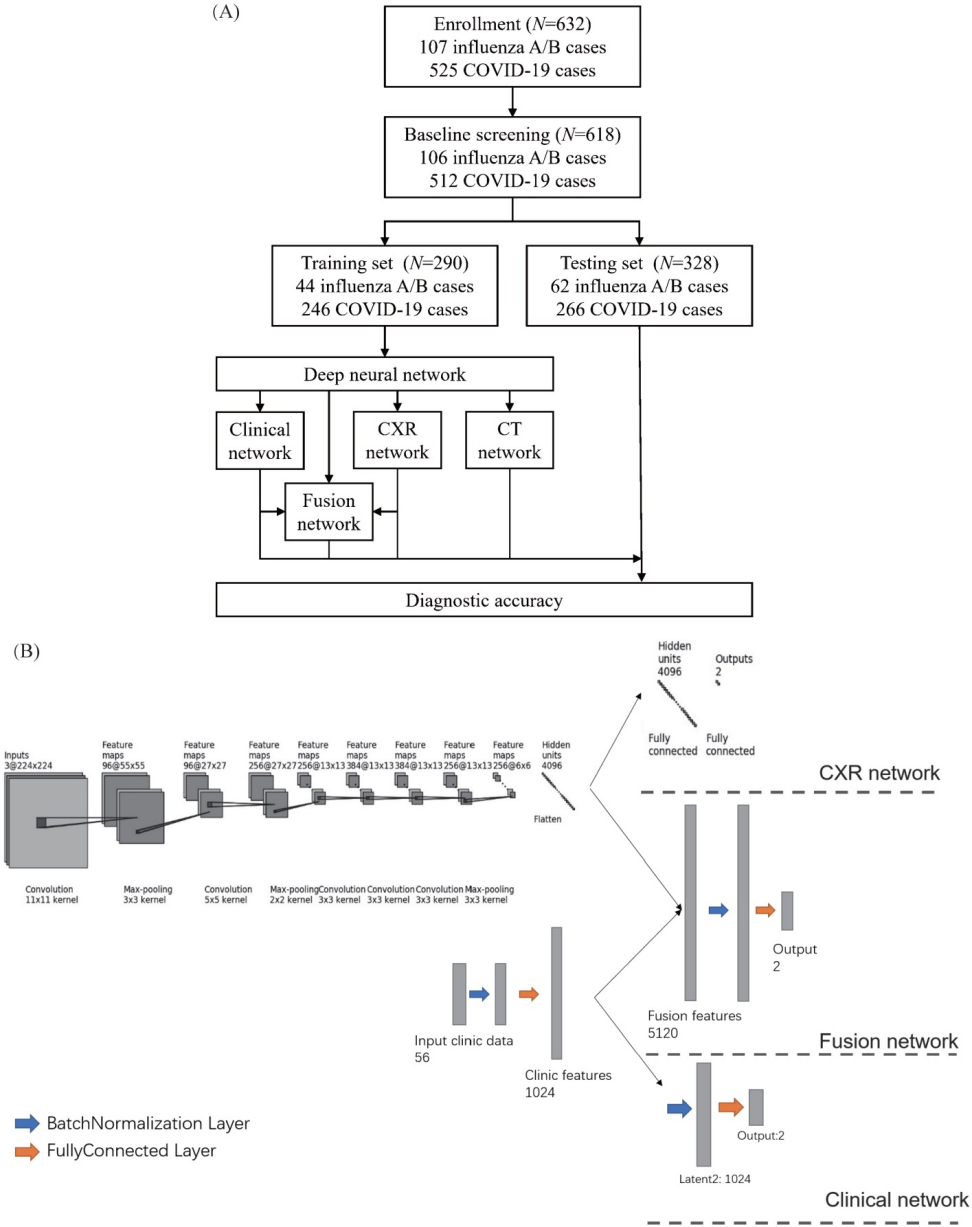
** Development of the classifier for differentiating coronavirus disease 2019 (COVID-19) from influenza A/B and structure of the deep neural network (DNN).** (A) A total of 525 patients with COVID-19 and 107 patients with influenza A/B were enrolled and separated into a training set of 290 cases and a test set of 328 cases after exclusion. A DNN was applied for feature extraction, selection, classification. The proposed fusion network, clinical network, chest x-ray (CXR) network and computed tomography (CT) network were established for final diagnosis. (B)The combined network system has two input streams: image data and clinical data. The two kinds of data are processed by two streams of deep neural layers, which are ultimately concatenated. When processing CXR image or clinical data only, the other one data stream is removed.

The retrospective study analyzed 525 patients with nucleic acid-confirmed COVID-19 and 107 patients with nucleic acid-confirmed influenza A/B pneumonia who were admitted to Wuhan Tongji Hospital and Second Affiliated Hospital Zhejiang University School of Medicine from January 2017 to June 2020. Since not all the patients underwent CXR, we used the chest CT localizer scan as a surrogate of a standard CXR. We excluded the patients with incomplete clinical data. The final cohort included 106 patients with influenza A/B epidemic viral pneumonia and 512 COVID-19 cases. We divided the cohort randomly into a training set of 290 cases (44 influenza A/B and 246 COVID-19) and a test cohort of 328 cases (62 influenza A/B and 266 COVID-19) (Figure 1A). The division of subset was totally random with simple random sampling principles. Their detailed demographic characteristics are listed in Table 1. The baseline clinical features, multi-stage chest CT and localizers, course of the disease, severe events, and interventions (drugs and supportive therapies) were collected from all patients. This retrospective study was approved by the ethics committees of the participating hospitals.

### Data Augmentation

Since a deep network should be trained on sufficient data and we enrolled fewer influenza cases than COVID-19 cases, data augmentation was done to produce more training cases. This process is widely used in deep learning and has proved useful for improving accuracy, especially when the number of cases is small or unbalanced. We firstly manually segment the lung areas because the localizer scan be totally unaligned. After segmentation, the image patches were resized to 256×256, and then random rotation (for -15~15 degree), scale (to 0.8~1.2 of the raw size), and transmit (1.0~1.1 of the raw size) were performed to augment our cases. Then we cropped a 224×224 patch from the augmented patches, and Gaussian noise was also randomly added to the training samples. Using the torchvision toolbox of Pytorch, the augmentation was done automatically in the training process and a sampler is introduced to make sure that influenza cases and COVID-19 cases are in the same amount in every batch.

### Deep Neural Network

A deep neural network (DNN) is a powerful machine-learning tool. Feature extraction, selection, and classification can be all formatted as neural layers using a DNN. To merge clinical patterns with CXR images, which combines clinical vectors with images, the proposed DNN input the clinical vectors and CXR images separately (Figure 1B). The CXR images were input as matrixes of gray values and convolutional construction extracted representative features from the images. The shallow layers focused on structural features, such as the shape, edge, and texture of lesions. The deep layers mined targeted semantic information, such as the presence or absence of a lesion and non-severe or severe grades of disease. We used Alexnet 21, a widely used deep network, for the CXR processing. For the clinical information, every element of the vector has different dimensionality and could be associated with every other element. We used a fully connected layer and a batch normalization layer to extract deep clinical features. The deep features derived from the CXR were then concatenated with the clinical features, resulting in fused features. Another fully connected layer and batch normalization layer were used to compute the final output, the combined diagnosis. The proposed fused network can be conveniently degraded into a clinical network or a CXR network by removing the other, as shown in Figure 1B. The DNN parts were all implemented based on Pytorch tools 22. We also evaluated the diagnostic performance of chest CT, which has proven to be a valuable tool for diagnosing COVID-19. Here, we used 3D densenet 23 as the CT diagnosis network. Since our cases are unbalanced in categories, we use number-balanced weights to keep loss function sensitive to both categories. Cross entropy of each category was multiplied by weights. Mathematically,


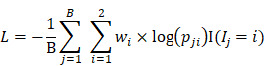


in which 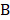 is batch size and 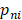 is the softmax-probability of case 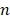 and category 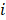. 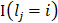 is indicative function which is 1 when the equitation 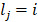 is true otherwise 0. 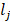 is the ground-truth label of category of case *j.* 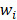 is the weight of category 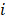 is computed by 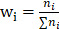.

### Statistical Analysis

In addition to the deep learning method, basic statistical analyses were performed. For univariate analyses, *t*-tests were used to compare 56 clinical features and demographic information. For multivariate analysis, we used the trained parameters identified from the clinical part of our deep learning network (Figure 1B) to compute the relative coefficients, which indicate the importance of each feature (Table S1). The area under the receiver operating curve (AUC), sensitivity, specificity, and negative and positive predictive values were also computed.

## Results

### Univariate and Multivariate Analysis of Clinical Features

We identified different patterns of clinical features for COVID-19 and influenza (Table 1). Fever, nasal congestion, sore throat, pharyngeal congestion, and productive sputum were common in both, while septic shock was more common in influenza and diarrhea was a salient symptom in COVID-19. Underlying lung disease and an impaired immune system were significantly associated with influenza, but not COVID-19, indicating that the entire population is susceptible to COVID-19, while influenza virus pneumonia is prone to affect specific patients. In influenza, the white blood cell count, C-reactive protein (CRP), procalcitonin, and bilirubin were increased, while in COVID-19 the number of platelets was significantly increased.

Table 2 shows the coefficients of each element derived from the multivariate model. Procalcitonin, urea nitrogen, and CRP were negatively related to COVID-19, as were fever and underlying lung disease. Conversely, conjunctival congestion, disseminated intravascular coagulation, obesity, and rhabdomyolysis were positively related to COVID-19.

### DNN Performances of Classifiers

**Figure 2:**
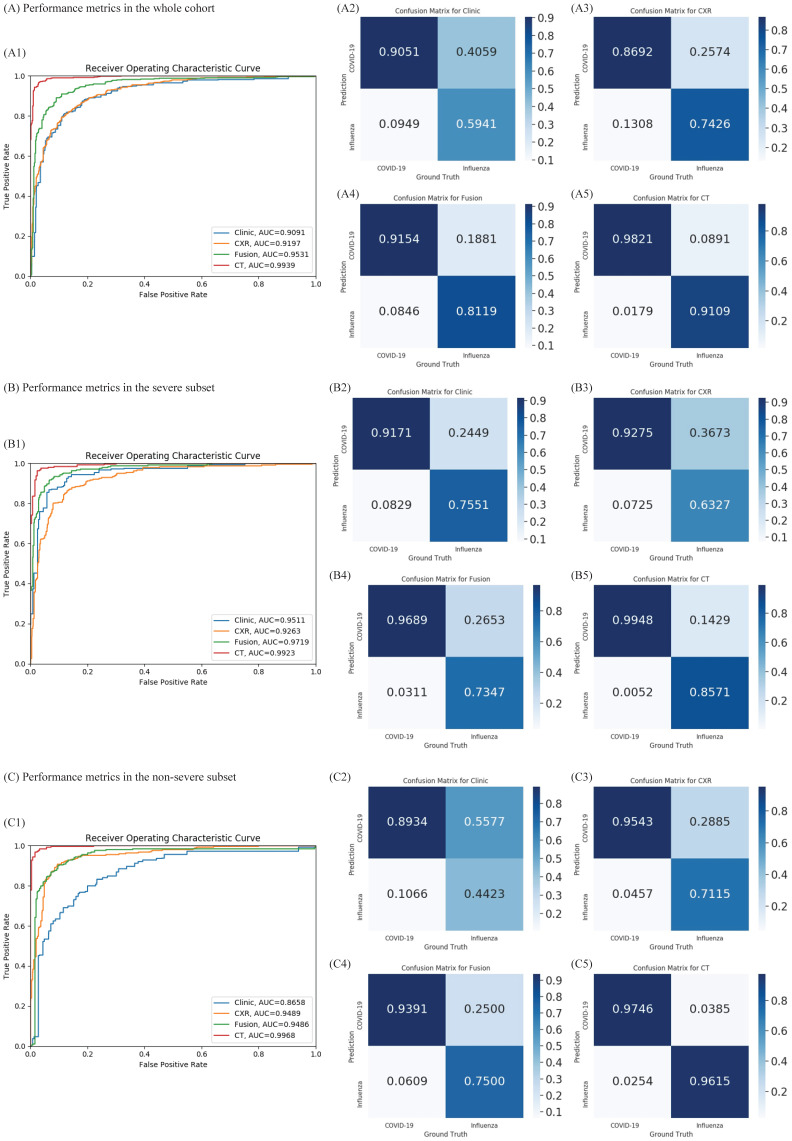
** Diagnostic performance of the proposed classifiers in the whole cohort, non-severe subset, and severe subset.** (A) Diagnostic performance in the whole cohort. According to the receiver operating characteristic (ROC) curves of our proposed method in the whole cohort (A1), combining the chest x-ray (CXR) and clinical data (green) improves the performance compared to both individually (blue and orange). A2-A5: Confusion metrics for clinical only (A2), CXR only (A3), combined (A4), and computed tomography (CT) (A5). Both the CXR and clinical data can diagnose coronavirus disease 2019 (COVID-19) and influenza. While the accuracy for diagnosing influenza using clinical features is relatively low and that for COVID-19 using CXR is lower, combining the clinical features and CXR improves both. (B) Diagnostic performance in the non-severe subset. As shown in the ROC curves for the non-severe subset (B1), clinical data (blue) perform better than chest x-ray (CXR) (orange). B2-B5: confusion metrics for clinical only (B2), CXR only (B3), combined (B4), and CT (B5) in non-severe patients. Combining the CXR and clinical data improves the diagnostic accuracy of COVID-19; although the diagnostic accuracy for influenza is slightly lower than with the clinical features only, the overall area under the curve is improved in the combined method. (C) Diagnostic performance in the severe subset. As presented in the ROC curves for the severe subset (C1), the diagnostic accuracy of CT outperformed the clinical feature or CXR. The area under the curve of the combined method is no better than for CXR only (*p* = 0.46). C2-C5: The confusion metrics for clinical only (C2), CXR only (C3), combined (C4), and CT (C5). AUC: area under the receiver operating curve.

**Figure 3:**
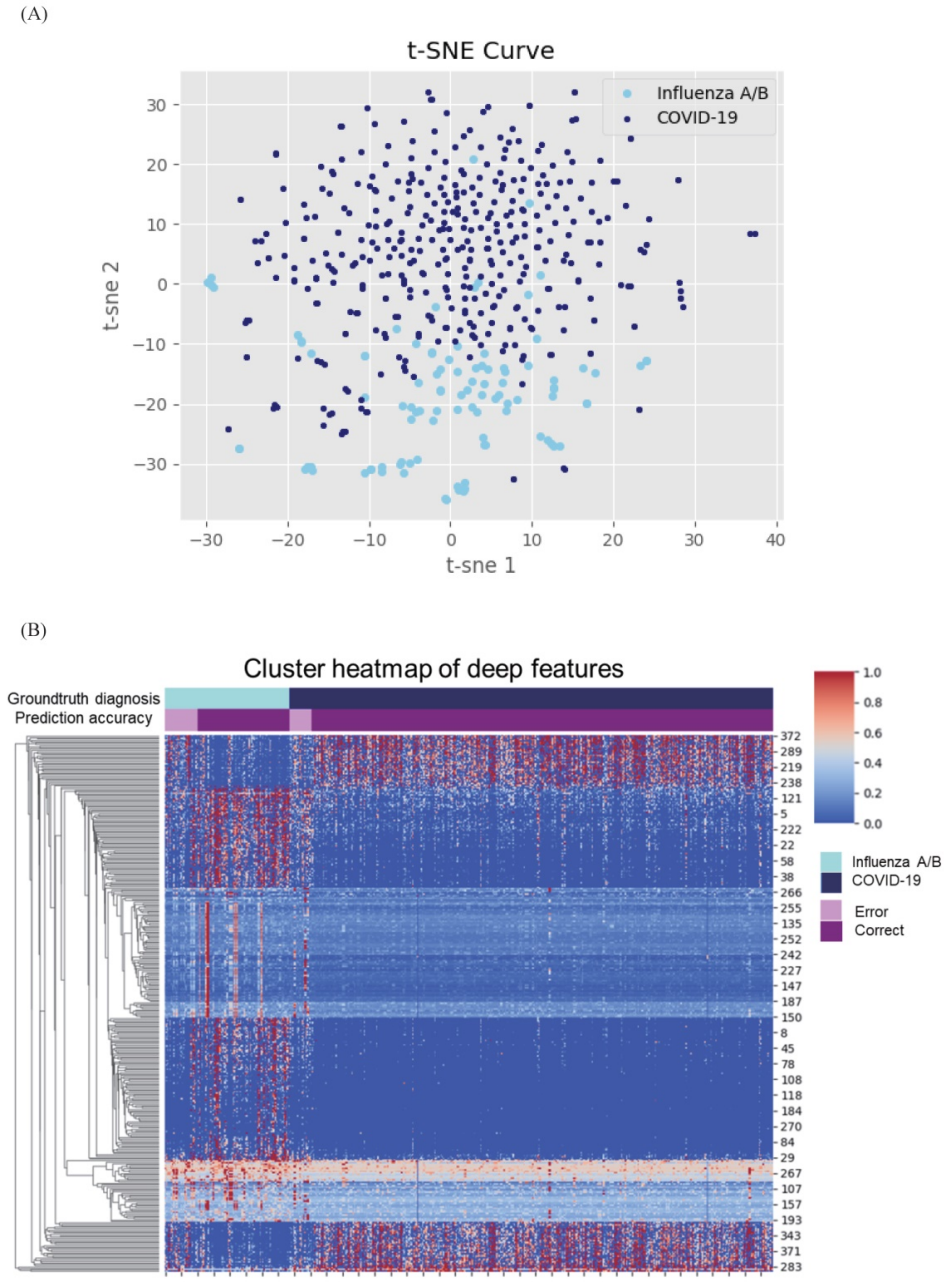
** Cluster heatmap and principal component analysis (PCA) of deep features in coronavirus disease 2019 (COVID-19) and influenza.** (A) Cluster heatmap of deep features in COVID-19 and influenza. Heatmap visualized most predominant 500 deep features among 5120 features and showed clear differences between individuals with COVID-19 and influenza A/B. (B) PCA of deep features in COVID-19 and influenza. The deep features separate COVID-19 from influenza A/B along the principal component.

Given the difference in clinical manifestations shown above, we first constructed a classifier using clinical features (Figure 2A). The AUC was 0.909 (95% CI 0.891-0.914) with a sensitivity of 90.5% and specificity of 59.4% in the test cohort. Next, we explored the diagnostic value of CXR and the validated AUC was 0.919 (95% CI 0.909-0.930) with a sensitivity of 86.9% and specificity of 74.2%. Separately, the clinical features and CXR performed comparably, but less than satisfactorily. When we combined the two, the AUC was increased to 0.952 (95% CI 0.944-0.960), with an improved sensitivity of 91.5% and notably specificity of 81.2%. Heatmap visualized top ranked 500 deep features among 5120 features and showed largely consistent differences between individuals with COVID-19 and influenza A/B (Figure 3A). Principal component analysis (PCA) double confirmed that COVID-19 and influenza A/B is a predominant source of variation in the dataset (Figure 3B). Since our method output possibility scores for cases, threshold points can be selected as cut-offs according to clinical needs. We also compared the diagnostic power of chest CT and our combined clinical-CXR modality. This showed that chest CT had a numerically higher AUC of 0.994 (95% CI 0.993-0.997), indicating that our combined modality is sufficiently accurate and capable of rapid screening.

### DNN Performance in severe and non-severe subgroups

Furthermore, we tested our rapid screening classifier (combined modality) in severe and non-severe subgroups (Figure 2B, 2C). In non-severe cases, our rapid screening classifier had an AUC of 0.971 (95% CI 0.964-0.980) with a sensitivity of 96.9% and specificity of 73.2%. The CXR classifier had an AUC of 0.926 (95% CI 0.914-0.941) with a per-exam sensitivity of 92.7% and specificity of 63.2%. The CT-based classifier had a per-exam sensitivity of 99.5% and specificity of 85.5%, with an AUC of 0.992 (95% CI 0.989-0.995). Our rapid screening classifier was as good as the CT-based classifier in the non-severe subgroup.

For severe cases, the observations differed. The CXR classifier had an AUC of 0.949 (95% CI 0.937-0.963), and combining the clinical features with the CXR characteristics failed to improve its efficacy (AUC 0.948 *vs*. 0.949, *P* = 0.452). In comparison, the AUC of the CT classifier was 0.996 (95% CI 0.995-0.998), which was comparable to the efficacy observed in the non-severe subgroup.

### The superiority of AI system to pulmonary physicians

**Figure 4:**
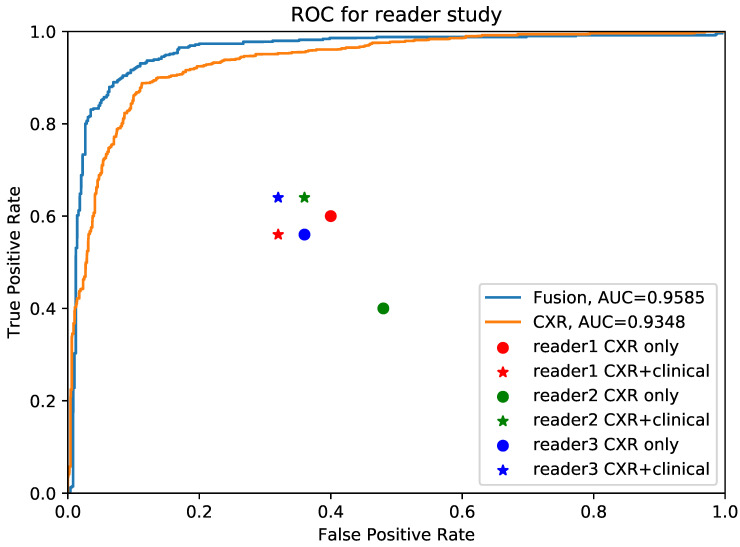
** Comparison between pulmonary physicians and artificial intelligence (AI) system.** The blue line is the receiver operating characteristic (ROC) curve of proposed AI system using fused clinical and chest x-ray (CXR) data, while the yellow one is the performance for CXR only. The round points are readers' results using only CXR and the star points are performances of pulmonary physicians using clinical data together with images.

We further conducted validation study which compared the diagnostic accuracy between AI system and 3 experienced pulmonary physicians (Figure 4). 50 cases, consisting of 25 COVID-19 individuals and 25 influenza individuals, were randomly selected. All readers were asked to read CXR independently without any clinical information in the first round, and to read with combined CXR and clinical information in the second round. The results showed that the average diagnostic accuracy of CXR for pulmonary physicians with and without clinical information was 0.467 and 0.473, respectively. The average reading time was 25 minutes. By contrast, the diagnostic AUC for AI system using CXR alone and CXR plus clinical information was 0.935 and 0.958, respectively, and the processing time was only 0.2 second.

## Discussion

We developed a rapid screening classifier to distinguish COVID-19 from influenza A/B pneumonia constructed using clinical and CXR features. It not only had comparable efficacy to chest CT but was also efficient, economical, and radiation safe. Of importance, for non-severe cases, the classifier combining clinical and CXR features had satisfactory efficacy, with an AUC of 0.9719. As most patients in the early stage of COVID-19 have mild illness, this is in line with our vision that the combined classifier is an ideal rapid screening tool. We also confirmed the value of chest CT in the diagnosis of COVID-19, especially its critical role in severe cases. However, the combined classifier based on clinical features and CXR remains a reliable alternative for screening severe COVID-19 when CT is not feasible for various reasons.

Our study revealed different patterns of symptoms in influenza and COVID-19. First, patients with COVID-19 pneumonia experienced less fever and had lower body temperatures than the patients with influenza, which indicates that patients with COVID-19 pneumonia can be asymptomatic. It is important that any screening system identify asymptomatic infectors to prevent them from turning into super-spreaders or severe cases. Our rapid screening classifier fits this role perfectly during the COVID-19 pandemic. Second, diarrhea was again found to be a typical symptom of COVID-19. Angiotensin-converting enzyme II (ACE2), which was highly expressed in both lung type II alveolar cells and gastrointestinal enterocytes, was proven to be the cell receptor of the novel SARS Cov-2 24. Therefore, diarrhea should be regarded as a warning sign for SARS CoV-2 infection 25, 26. Third, a higher percentage of influenza patients had an impaired immune system and underlying lung diseases than did COVID-19 patients. It could be explained by blunted T and NK cell amount and function in influenza patients resulted in greater susceptibility 27-29. Conversely, the entire population is susceptible to SARS CoV-2, but older patients with comorbidities need greater vigilance regarding worsening disease.

In addition, elevated D-dimer was significantly correlated with COVID-19, suggesting a sustained hypercoagulable state during SARS CoV-2 infection. In concert, a high incidence of thromboembolic events has been described in COVID-19, especially in critically ill individuals 30-33. It is still not clear whether SARS-CoV-2 attacks vascular endothelial cells directly. However, SARS CoV-2 infection may predispose to thromboembolism 34, in which elevated levels of proinflammatory factors (including IL-6, GM-CSF, IL1B, and IFN-γ) may play a role 35-38. COVID-19 infection promotes the transformation of pathogenic T lymphocytes and induces inflammatory monocytes to express IL-6 and accelerate inflammation 38. Hence, a coagulation cascade may be activated by a cytokine storm 32.

The lower incidence of septic shock in COVID-19 was in line with the finding that up to 76% of the COVID-19 cohort was culture-negative for bacteria and fungi 24. The hypothesis of virus sepsis and severe COVID-19 is a topic of lively debate 32. Immune response disorders characterized by cytokine storms may be positively involved in the pathogenic mechanism of viral sepsis 39. The cytokine storm induced by invasion of the novel coronavirus causes diffuse lung damage and systematic inflammation, leading to multiple organ failure and viral sepsis 39. Interleukin 6 and GM-CSF are two key triggers in cytokine storms 40. Application of cytokine-modulatory therapy, especially anti-IL-6 agents, is expected to improve the prognosis of severe COVID-19.

We used CT localizer scans as surrogates of a standard CXR. Localizer scans are physically equivalent to an x-ray, although differences remain. Localizer scans can be presented as coronal and sagittal scans of patients in a supine position, although their parameter adjustment is not as precise as for x-rays, leading to lower-quality images containing less radiological information. Therefore, we may have underestimated the value of CXR. In addition, localizers usually cover a wider view than CXR and the additional imaging, such as of ventilators, may result in artificial effects. To overcome these issues, we cropped out the lung areas of every scan manually to force the system to focus on lung area when making diagnostic decisions. Although viral pneumonias usually have similar imaging characteristics, there are still different radiological patterns between COVID-19 and influenza, in either CT or x-rays. The predominant pattern in COVID-19 is characterized by ground-glass opacities and consolidation opacities with a peripheral distribution, while the typical radiological findings of influenza are diffuse ground-glass opacities and small nodules with more central locations 41-44. In the bronchovascular area, a crazy-paving pattern is observed more often in COVID-19 and often indicates a poor prognosis 44, 45. Pleural effusions are more common in influenza, while pleural thickening may occur in COVID-19 44, 46, 47.

There are several limitations to our study. First, the numbers of cases of COVID-19 and influenza were not balanced, which may increase the overfitting risk. The prediction of all cases in the category with the greatest numbers (COVID-19 in our experiments) can also yield good accuracy performance, with high sensitivity, but low specificity. Our model overcame this drawback by adding number-balanced weights for loss function and augmenting the size of the influenza category with fewer patients, resulting in high sensitivity and specificity, and successfully eliminating the influence of unbalanced numbers. Second, because this study examined retrospective cohorts, larger prospective validation cohorts are warranted in the future.

In conclusion, we devised a rapid screening classifier constructed using clinical and CXR features to distinguish COVID-19 from influenza A/B pneumonia. The classifier was efficient, economical, and radiation safe. Our combined classifier may be an ideal rapid screening tool during the COVID-19 pandemic.

## Supplementary Material

Supplementary table S1.

## Figures and Tables

**Table 1:** Clinical characteristics and laboratory findings of patients with A/B influenza or COVID-19

Characteristics	A or B influenza	COVID-19	*P-*value
Male sex - no. (%)	39 (36.79%)	268 (52.34%)	0.0035
Median age (IQR) - year	59.8 (48.5-72.0)	60.8 (53.0-70.0)	0.5301
Fever on/after admission			
Fever on admission - no. (%)	81 (76.42%)	146 (28.52%)	<0.0001
Chills on admission - no. (%)	16 (15.09%)	68 (13.28%)	0.6593
Highest temperature on admission - °C	38.27 (0.94)	37.60 (1.05)	<0·0001
Fever during hospitalization - no. (%)	73 (68.87%)	260 (50.78%)	0.1433
Chills during hospitalization - no. (%)	17 (16.04%)	37 (7.23%)	0.0011
Highest temperature during hospitalization - °C	38.24 (0.84)	37.61 (0.76)	<0·0001
**Symptoms - no. (%)**			
Conjunctival congestion	0 (0.00%)	0 (0.00%)	0.5200
Nasal congestion	13 (12.26%)	6 (1.17%)	<0·0001
Cough	96 (90.57%)	386 (75.39%)	0.0006
Expectoration	91 (85.85%)	222 (43.36%)	<0·0001
Sore throat	28 (26.42%)	28 (5.47%)	<0·0001
Fatigue	48 (45.28%)	197 (38.48%)	0.1747
Dyspnea/Shortness of breath	58 (54.72%)	245 (47.85%)	0.2111
Hemoptysis	10 (9.43%)	11 (2.15%)	0.0003
Nausea/Vomit	7 (6.60%)	70 (13.67%)	0.0397
Headache	17 (16.04%)	57 (11.13%)	0.1754
Diarrhea	8 (7.55%)	133 (25.98%)	<0·0001
Myalgia	26 (24.53%)	102 (19.92%)	0.3087
**Signs of infection - no. (%)**			
Pharyngeal congestion	11 (10.38%)	8 (1.56%)	<0·0001
Enlarged tonsils	1 (0.94%)	0 (0.00%)	0.1544
Enlarged lymph node	2 (1.89%)	0 (0.00%)	0.0124
Rash	3 (2.83%)	0 (0.00%)	0.0010
**Complication - no. (%)**			
Septic shock	9 (8.49%)	5 (0.98%)	<0·0001
Acute respiratory distress syndrome	12 (11.32%)	28 (5.47%)	0.0356
Acute kidney injury	10 (9.43%)	15 (2.93%)	0.0029
Disseminated intravascular coagulation	0 (0.00%)	6 (1.17%)	0.3791
Rhabdomyolysis	0 (0.00%)	8 (1.56%)	0.1574
**Underlying disease - no. (%)**			
Hypertension	36 (33.96%)	184 (35.94%)	0.6713
Heart disease	11 (10.38%)	48 (9.38%)	0.7979
Diabetes	12 (11.32%)	81 (15.82%)	0.2197
Obesity	6 (5.66%)	4 (0.78%)	0.0014
Lung disease	22 (20.75%)	28 (5.47%)	<0·0001
Kidney disease	5 (4.72%)	8 (1.56%)	0.0395
Liver Disease	6 (5.66%)	7 (1.37%)	0.0109
Cancer	5 (4.72%)	18 (3.52%)	0.6224
Impaired immune system	9 (8.49%)	6 (1.17%)	<0·0001
**Outcome - no. (%)**			
Critically ill	44 (41.51%)	228 (44.53%)	0.5439
Admission to ICU	38 (35.85%)	8 (1.56%)	<0·0001
Mechanical ventilation	31 (29.25%)	24 (4.69%)	<0·0001
Death	7 (6.60%)	5 (0.98%)	0.0003
**Laboratory findings**			
White blood cell count - ×10^9^/L	8.31 (4.93)	6.20 (2.89)	<0·0001
Lymphocyte count - ×10^9^/L	1.07 (0.59)	1.16 (1.01)	0.4042
Platelet count - ×10^9^/L	183.18 (84.43)	243.68 (99.65)	<0·0001
Hemoglobin - g/L	122.85 (21.85)	126.62 (14.98)	0.0312
C-reactive protein - mmol/L	83.09 (66.32)	49.91 (54.24)	<0·0001
Procalcitonin - ng/mL	3.69 (11.52)	0.98 (1.19)	<0·0001
Serum sodium - mmol/L	138.18 (5.19)	138.09 (9.05)	0.9232
Serum potassium - mmol/L	3.90 (0.63)	4.87 (9.42)	0.2887
Serum chlorine - mmol/L	101.42 (5.44)	99.95 (5.99)	0.0200
Serum calcium - mmol/L	2.03 (0.30)	2.98 (11.69)	0.4045
Lactate dehydrogenase - U/L	364.10 (233.64)	315.13 (135.76)	0.0036
Alanine aminotransferase - U/L	42.59 (34.17)	40.72 (121.47)	0.8755
Aspartate aminotransferase - U/L	59.02 (76.76)	42.69 (150.26)	0.2768
Bilirubin - mmol/L	12.77 (7.36)	9.63 (4.89)	<0·0001
Creatine Kinase - U/L	426.49 (1816.07)	240.37 (125.51)	0.0224
Creatinine - μmol/L	95.59 (108.63)	73.66 (51.45)	0.0017
Urea Nitrogen - mmol/L	7.61 (7.30)	5.20 (9.68)	0.0158
D-dimer - mg/L	306.18 (692.31)	131.41 (476.43)	0.0017
Activated partial thromboplastin time - s	42.63 (9.66)	40.08 (5.70)	0.0003
Prothrombin time - s	14.80 (6.90)	14.18 (2.22)	0.0945

**Footnote:** IQR: interquartile range.

**Table 2:** Differential diagnostic efficacy of four classifiers in the whole cohort, non-severe subset, and severe subset

Classifier	AUC	Sensitivity	Specificity	NPV	PPV
**Whole cohort**					
Clinic only	0.9091 (0.8918-0.9145)	0.9046 (0.8846-0.9197)	0.5941 (0.6173-0.6914)	0.5985 (0.5652-0.6344)	0.9080 (0.8947-0.9208)
CXR only	0.9197 (0.9090-0.9302)	0.8692 (0.8539-0.8805)	0.7425 (0.7037-0.7805)	0.5932 (0.5607-0.6316)	0.9284 (0.9167-0.9420)
Proposed fusion	0.9524 (0.9443-0.9608)	0.9154 (0.9055-0.9299)	0.8119 (0.7763-0.8434)	0.7139 (0.6778-0.7500)	0.9492 (0.9400-0.9600)
CT	0.9946 (0.9932-0.9970)	0.9818 (0.9773-0.9872)	0.9112 (0.8902-0.9359)	0.9284 (0.9103-0.9500)	0.9771 (0.9712-0.9838)
**Non-severe subset**					
Clinic only	0.9514 (0.9408-0.9629)	0.9172 (0.9013-0.9342)	0.7551 (0.7105-0.8056)	0.6985 (0.6486-0.7500)	0.937 (0.9216-0.9530)
CXR only	0.9263 (0.9140-0.9413)	0.9275 (0.9145-0.9430)	0.6327 (0.5814-0.6923)	0.6876 (0.6364-0.7500)	0.9082 (0.8910-0.9255)
Proposed fusion	0.9719 (0.9648-0.9808)	0.9689 (0.9605-0.9806)	0.7347 (0.6774-0.7805)	0.8588 (0.8182-0.9118)	0.9343 (0.9193-0.9494)
CT	0.9923 (0.9892-0.9954)	0.9948 (0.9934-1.0000)	0.8571 (0.8158-0.8974)	0.9765 (0.9677-1.0000)	0.9645 (0.9554-0.9750)
**Severe subset**					
Clinic only	0.8666 (0.8489-0.8925)	0.8934 (0.8782-0.9146)	0.4423 (0.3846-0.5000)	0.5230 (0.4688-0.5882)	0.8590 (0.8415-0.8802)
CXR only	0.9494 (0.9374-0.9635)	0.9543 (0.9308-0.9627)	0.7115 (0.7027-0.7500)	0.7865 (0.7250-0.8421)	0.9357 (0.9202-0.9506)
Proposed fusion	0.9487 (0.9378-0.9629)	0.9391 (0.9255-0.9548)	0.7500 (0.7073-0.8000)	0.7654 (0.7179-0.8158)	0.9349 (0.9226-0.9494)
CT	0.9968 (0.9955-0.9989)	0.9746 (0.9679-0.9871)	0.9615 (0.9474-0.9778)	0.9107 (0.8810-0.9512)	0.9897 (0.9868-0.9938)

**Footnote:** AUC: area under the receiver operating curve; NPV: negative predictive value; PPV: positive predictive value; CXR: chest X-ray; CT: computed tomography.
