# Supplementary material for: A rapid screening classifier for diagnosing COVID-19
Source: Int J Biol Sci. 2021 Jan 9;17(2):539–48. doi: 10.7150/ijbs.53982 (PMC7893593; doi:10.7150/ijbs.53982)
Supplement: Supplementary file 1 — Supplementary table S1. [file ijbsv17p0539s1.pdf]

Supplementary Materials

Table S1 Relative coefficients of each clinic feature in deep learning model.

Clinic features	Relative coefficients	Clinic features	Relative coefficients
Fever on admission	-0.072	Cough	-0.0068
Urea Nitrogen	-0.0707	Chills during hospitalization	-0.0019
Procalcitonin	-0.0632	Activated partial thromboplastin time	-0.0017
Lung disease	-0.0579	Myalgia	0.0002
C-reactive protein	-0.0571	Hypertension	0.0003
Liver Disease	-0.0513	Highest temperature during hospitalization	0.0004
White blood cell count	-0.0448	Heart disease	0.0023
Rash	-0.0445	Cancer	0.0035
Expectoration	-0.0439	Diabetes	0.0068
Sore throat	-0.0423	Aspartate aminotransferase	0.0081
Hemoptysis	-0.0389	Serum chlorine	0.0081
Septic shock	-0.0349	Pharyngeal congestion	0.0113
Impaired immune system	-0.0345	Chills on admission	0.0156
Fever during hospitalization	-0.0312	Enlarged tonsils	0.0162
Kidney disease	-0.0248	Serum potassium	0.0189
Prothrombin time	-0.023	Highest temperature on admission	0.02
D-dimer	-0.0224	Serum sodium	0.0249
Alanine aminotransferase	-0.0219	Headache	0.0267
Lymphocyte count	-0.0172	Acute kidney injury	0.0281
Creatinine	-0.0146	Nausea / Vomit	0.0283

Acute respiratory distress syndrome	-0.0115	Platelet count	0.0307
Hemoglobin	-0.0109	Rhabdomyolysis	0.0338
Serum calcium	-0.0102	Conjunctival congestion	0.0374
Nasal congestion	-0.0097	Disseminated intravascular coagulation	0.0377
Enlarged lymph node	-0.0095	Obesity	0.0436
Fatigue	-0.0088	Lactate dehydrogenase	0.0529

Footnote: Huger absolute value means more important in model, and minus value means negative correlation and vice versa.
